# Supplementary material for: Single-nuclei transcriptome analysis of Huntington disease iPSC and mouse astrocytes implicates maturation and functional deficits
Source: iScience. 2022 Dec 6;26(1):105732. doi: 10.1016/j.isci.2022.105732 (PMC9800269; doi:10.1016/j.isci.2022.105732)
Supplement: Document S1. Figures S1–S7 [file mmc1.pdf]

iScience, Volume *26*

Supplemental information

Single-nuclei transcriptome analysis of Huntington disease iPSC and mouse astrocytes implicates maturation and functional deficits

Andrea M. Reyes-Ortiz, Edsel M. Abud, Mara S. Burns, Jie Wu, Sarah J. Hernandez, Nicolette McClure, Keona Q. Wang, Corey J. Schulz, Ricardo Miramontes, Alice Lau, Neethu Michael, Emily Miyoshi, David Van Vactor, John C. Reidling, Mathew Blurton-Jones, Vivek Swarup, Wayne W. Poon, Ryan G. Lim, and Leslie M. Thompson

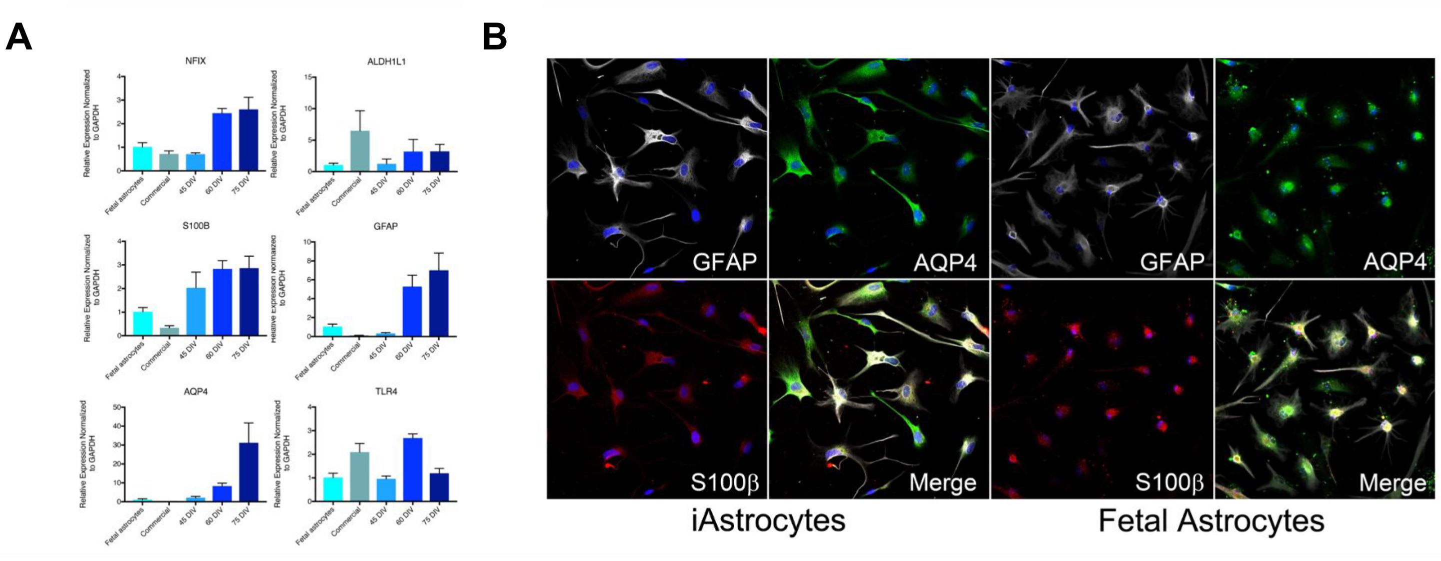

Figure S1. iAstro differentiation compared to other human astrocyte cell lines, related to Figure 1. (**A**) Quantitative PCR on astrocyte marker transcripts compared to human fetal and commercially available astrocytes from FUJIFILM Cellular Dynamics, Inc. (**B**) Immunocytochemistry of astrocyte marker proteins compared to human fetal astrocytes.

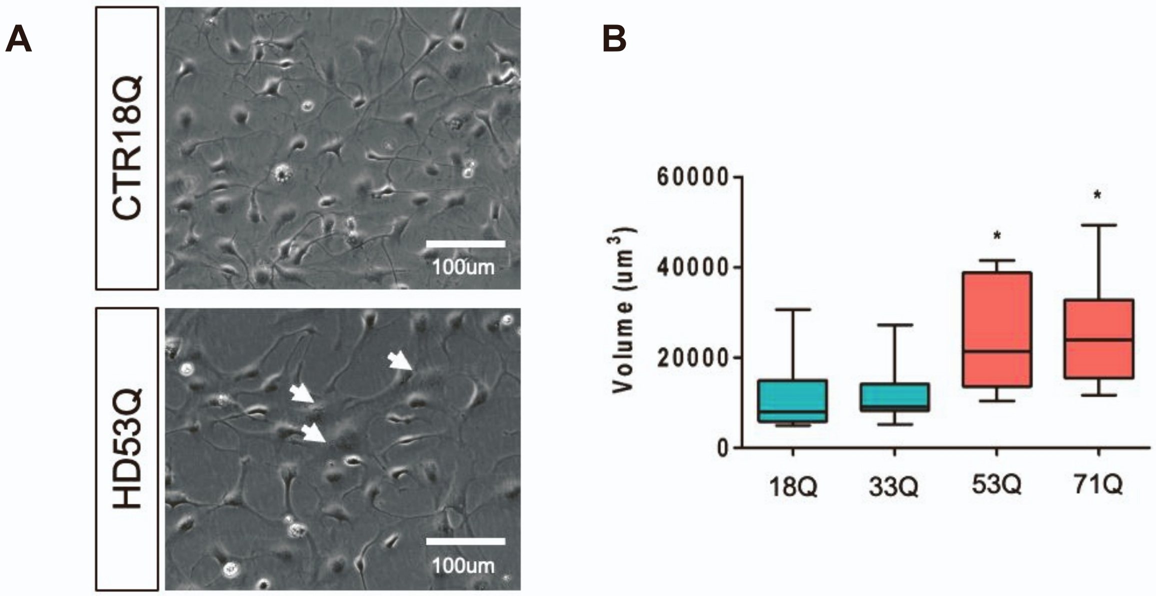

Figure S2. HD and control iAstro morphology differences, related to Figure 1. (**A**) Representative phase contrast images of unsorted day 60 iAstros morphology. HD iAstro white arrows highlight HD iAstros with enlarged cell bodies. (**B**) Quantification of cellular volume by confocal z-stacks of GFAP-positive iAstros at day 60 (one-way ANOVA: *p<0.05, n = 3 biological [differentiation] replicates per line). All error bars indicate mean ± standard error mean.

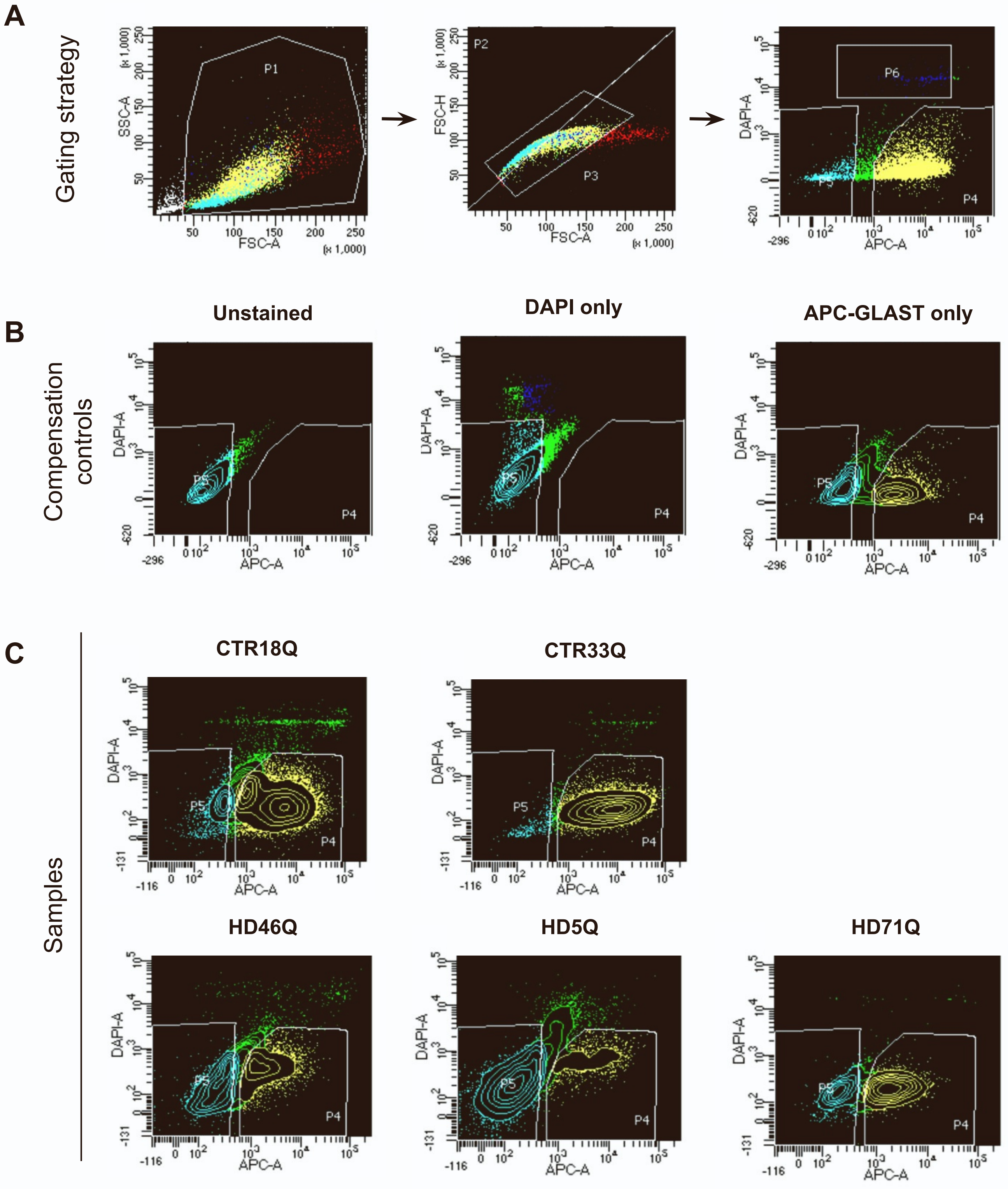

Figure S3. Representative GLAST FACS plots, related to Figure 1. (**A**) Gating strategy to remove cellular debris and select single cells. (**B**) Compensation controls for unstained cells and single-stains of DAPI only and APC-GLAST only. (**C**) Representative FACS plot for each day 60 iAstro cell line. Population 4 (P4; yellow) was collected and used for subsequent assays as GLAST-positive iAstros. Related to Figure 1.

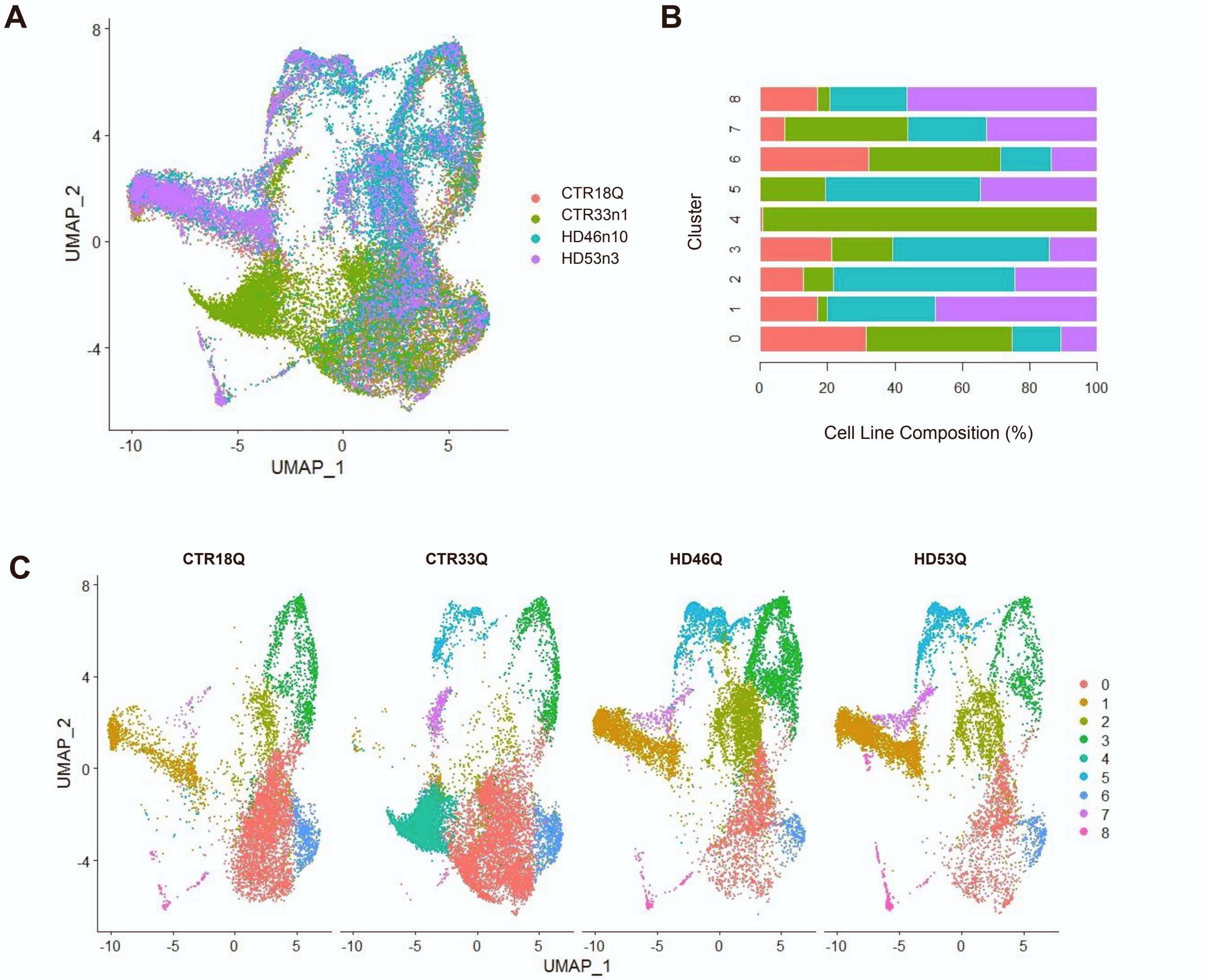

Figure S4. HD and control iAstro UMAPs and cluster breakdown by cell line, related to Figure 2.
(**A**) GLAST-positive iAstro snRNA-seq UMAP colored by cell line (control [CTR18Q, CTR33Q] n=2, HD [HD46Q, HD53Q] n=2). (**B**) GLAST-positive iAstro cell line composition across astrocyte clusters. Clusters 0, 4, and 6 are mostly composed of control cells, with cluster 4 primarily composed of CTR33n1 cells. Clusters 1, 2, 5, and 8 are mostly composed of HD cells. Clusters 3 and 7 are distributed fairly evenly across all cell lines. (**C**) GLAST-positive iAstro snRNA-seq UMAPs split by cell line (control [CTR18Q, CTR33Q] n=2, HD [HD46Q, HD53Q] n=2) and colored by cluster.

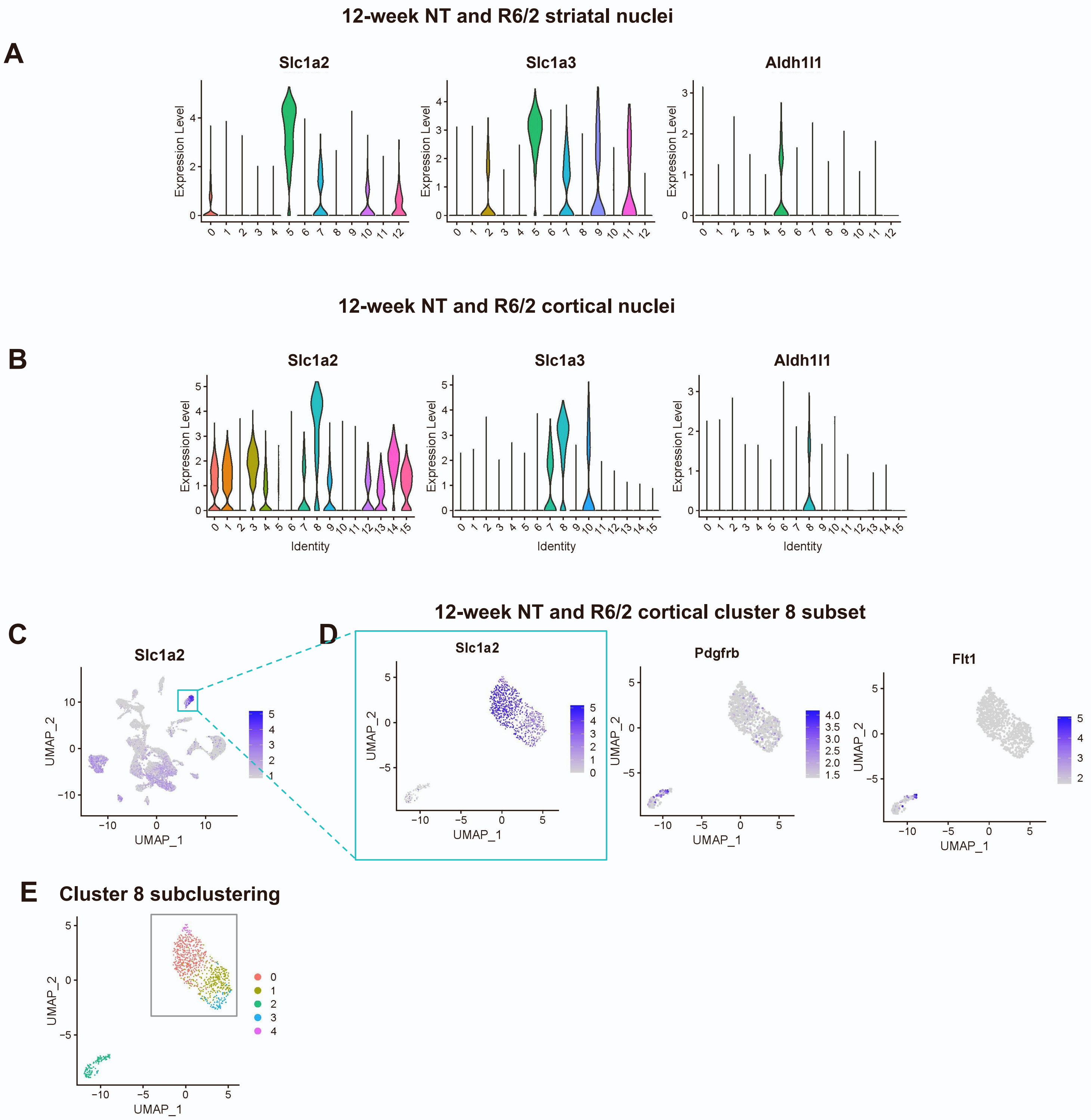

Figure S5. Astrocyte cluster selection for NT and R6/2 12-week striatal and cortical snRNAseq, related to Figures 3 & 4. (**A**) Violin plots of astrocyte markers depict striatal cluster 5 as the astrocyte cluster by highest astrocyte marker expression. (**B-D**) Violin plots (B) and UMAP visulization (C) of astrocyte markers depict cortical cluster 8 as the astrocyte cluster by highest astrocyte marker expression. (**D**) Subset of cortical cluster 8 with expression astrocyte markers and vascular markers shows multiple cell types. (**E**) Clustering analysis of cortical cluster 8 deomstrates subcluster 2 (green) contains significantly different gene expression compared to other subclusters. Subclusters 0, 1, 3, 4 (gray box) were subset and deemed the revised cortical astrocyte cluster for subsequent cortical analyses.

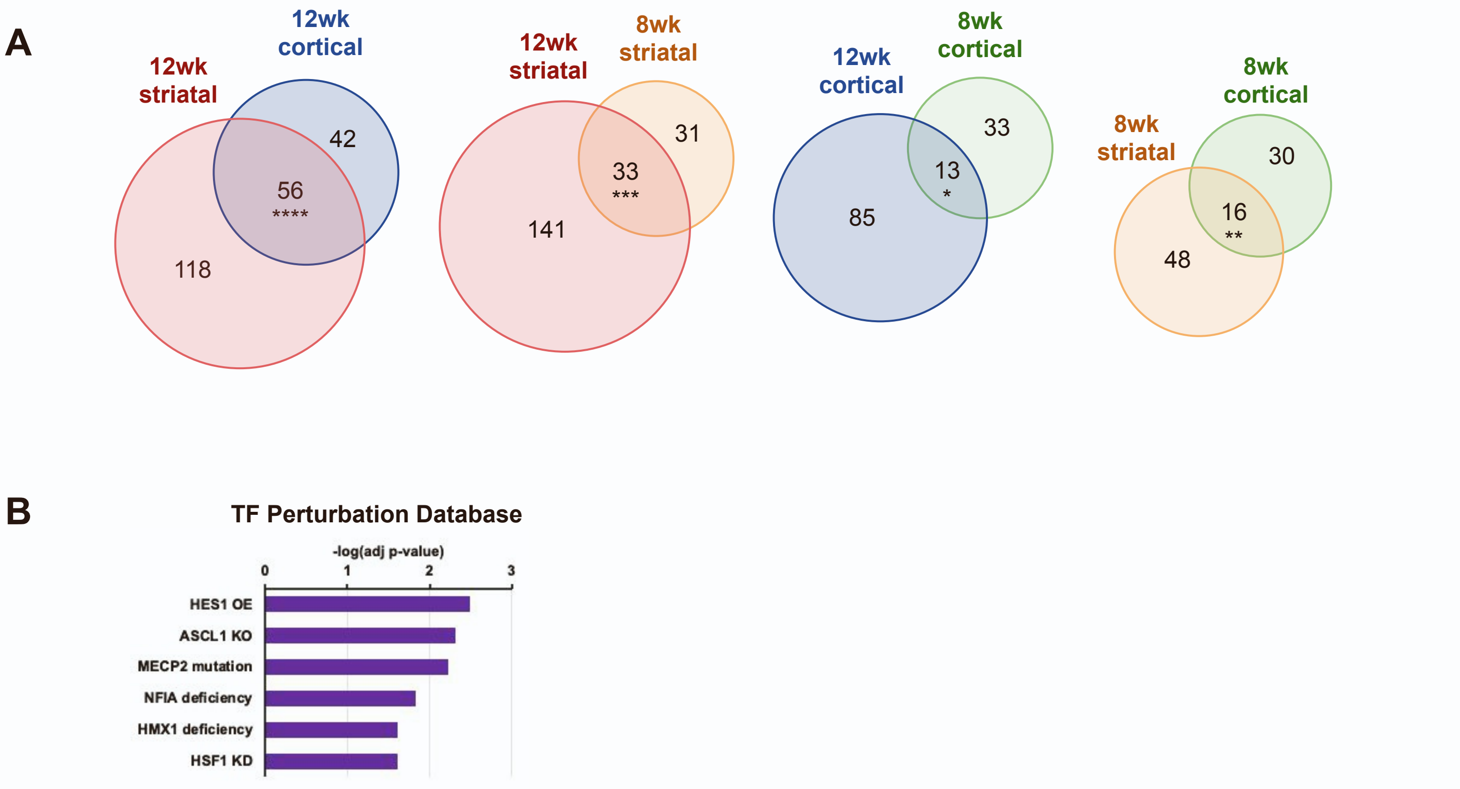

Figure S6. R6/2 astrocyte differentially expressed gene overlaps and transcription factor enrichment, related to Figure 5. (**A**) Venn diagrams of significant differentially expressed genes (DEGs) in R6/2 mouse astrocytes across time points and brain region (Exact hypergeometric probability calculated: using 56 DEG overlap for 12 week striatum [174 DEGs] vs 12 week cortex [98 DEGs] ****p < 7.190e-113, 33 DEG overlap for 12 week striatum [174 DEGs] vs 8 week striatum [64 DEGs] ***p < 6.427e-64, 13 DEG overlap for 12 week cortex [98 DEGs] vs 8 week cortex [46 DEGs] *p < 7.335e-25, 16 DEG overlap for 8 week striatum [64 DEGs] vs 8 week cortex [46 DEGs] **p < 2.281e-35 using 46,206 as the number of genes with nucleotide sequence data in the mouse genome database). (**B**) Overlap of R6/2 12wk striatal and 12wk cortical astrocyte DEGs predicted transcription factor regulation using Enrichr.

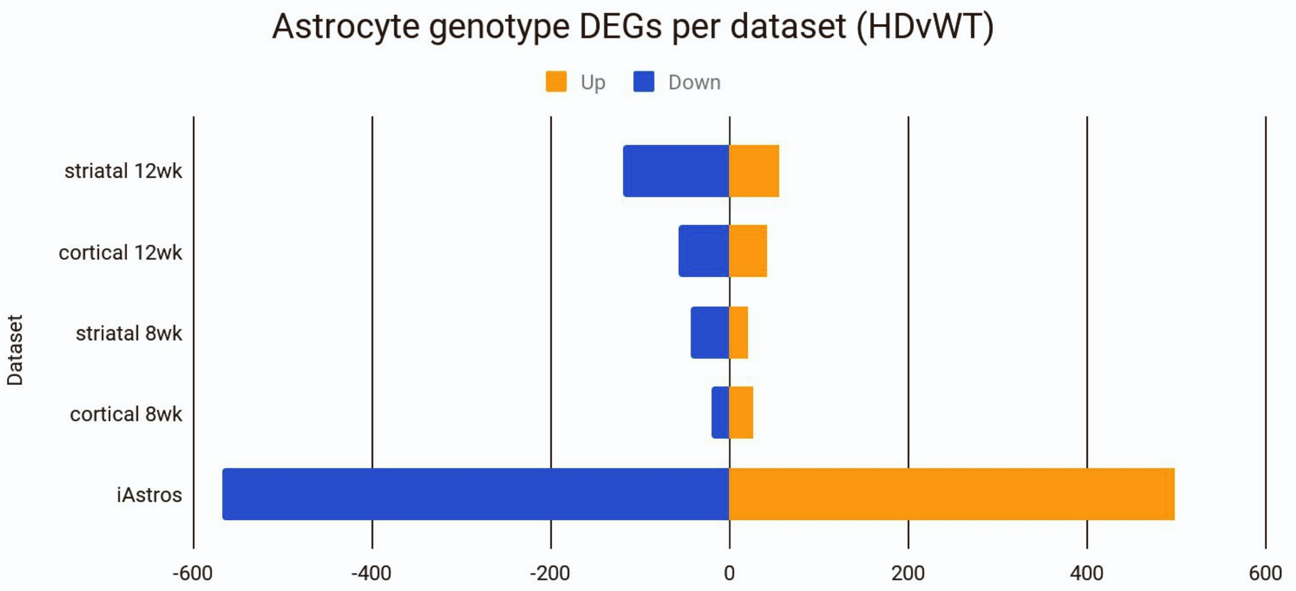

Figure S7. Genotype DEG counts, related to Figures 2-5. R6/2 astrocyte and HD iAstro significant differentially expressed gene count. Orange represents an upregulated (positive) gene and blue represents a (negative) downregulated gene count.
